# High–low Kelvin probe force spectroscopy for measuring the interface state density

**DOI:** 10.3762/bjnano.14.18

**Published:** 2023-01-31

**Authors:** Ryo Izumi, Masato Miyazaki, Yan Jun Li, Yasuhiro Sugawara

**Affiliations:** 1 Department of Applied Physics, Graduate School of Engineering, Osaka University, 2-1 Yamadaoka, Suita, Osaka 565-0871, Japan

**Keywords:** high–low Kelvin probe force microscopy, high–low Kelvin probe force spectroscopy, interface state density, Kelvin probe force microscopy, Kelvin probe force spectroscopy

## Abstract

The recently proposed high–low Kelvin probe force microscopy (KPFM) enables evaluation of the effects of semiconductor interface states with high spatial resolution using high and low AC bias frequencies compared with the cutoff frequency of the carrier transfer between the interface and bulk states. Information on the energy spectrum of the interface state density is important for actual semiconductor device evaluation, and there is a need to develop a method for obtaining such physical quantities. Here, we propose high–low Kelvin probe force spectroscopy (high–low KPFS), an electrostatic force spectroscopy method using high- and low-frequency AC bias voltages to measure the interface state density inside semiconductors. We derive an analytical expression for the electrostatic forces between a tip and a semiconductor sample in the accumulation, depletion, and inversion regions, taking into account the charge transfer between the bulk and interface states in semiconductors. We show that the analysis of electrostatic forces in the depletion region at high- and low-frequency AC bias voltages provides information about the interface state density in the semiconductor bandgap. As a preliminary experiment, high-low KPFS measurements were performed on ion-implanted silicon surfaces to confirm the dependence of the electrostatic force on the frequency of the AC bias voltage and obtain the interface state density.

## Introduction

With the recent miniaturization of semiconductor devices, understanding the physical and electrical properties of semiconductor devices, such as the dopant concentration, dopant distribution, and defect level distribution, at the nanoscale has become important. Among the physical properties of semiconductors, information on semiconductor interface states is particularly important. For example, in semiconductor devices such as field-effect transistors, the presence of semiconductor interface states is known to significantly affect device operation characteristics [1–3]. Therefore, direct observation of semiconductor surfaces with nanoscale spatial resolution will become even more important for understanding and controlling the effects of these properties on devices and for evaluating semiconductor device operation.

Kelvin probe force microscopy (KPFM) is known as a method that can measure the contact potential difference (CPD) between a tip and a sample with high spatial resolution [4–5]. KPFM is based on the detection of the electrostatic force between a tip and a sample using atomic force microscopy (AFM) [6–8]. CPD and topographic measurements have been performed on a variety of sample surfaces, including metals [9–10], semiconductors [11–14], and insulators [15–17]. When a semiconductor sample is measured by KPFM, the measured CPD is related to information about the semiconductor properties such as dopant density, surface charge, band bending, and interface state density [18]. In particular, previous studies of silicon substrates with different impurity concentrations measured by KPFM have shown that when the impurity concentration is very high (>10^16^ cm^−3^), surface band bending occurs, and the measured CPD approaches that of the intrinsic semiconductor [19]. Thus, since the CPD is strongly affected by the surface properties, accurate evaluation of the surface state and bulk impurity concentration requires a method that extracts only the surface potential effect due to interface states.

Recently, we proposed high–low Kelvin probe force microscopy (high–low KPFM) as a technique to solve the above problem [20–21]. High–low KPFM is a method for measuring the magnitude and direction of band bending due to interface states by applying low-frequency and high-frequency AC bias voltages between the tip and the sample with respect to the cutoff frequency *f*_c_ of carrier transport between the bulk and interface states and measuring the difference in CPD by KPFM. In high–low KPFM, frequency modulation (FM) KPFM (FM-KPFM) combined with FM-AFM is used to detect the tip–sample interaction force. FM-KPFM has several advantages, namely high sensitivity to the electrostatic force gradient, high detection sensitivity using a cantilever with a weak spring constant at the first resonance, ease of implementation in adding FM-AFM, and no need to enhance the bandwidth of the cantilever deflection sensor. FM-KPFM is used to apply an AC bias voltage at frequencies lower than the cutoff frequency *f*_c_ of carrier transport, and heterodyne FM-KPFM, based on the heterodyne effect (frequency conversion effect) between mechanical oscillation of the cantilever and electrostatic force oscillation, is used to apply an AC bias voltage at frequencies higher than the cutoff frequency *f*_c_ of carrier transport. To date, high–low KPFM has successfully visualized the surface band bending of pn-patterned silicon substrates [22]. However, in high–low KPFM, the CPD is compensated by a DC bias voltage. Hence, a certain DC voltage, determined by the CPD, is applied to the semiconductor sample. Therefore, the surface potential of the semiconductor is fixed at a certain energy, and only the surface state near the Fermi level of the surface is reflected in CPD measurements, making measurement of the energy distribution of the interface states within the bandgap difficult. Thus, a method for measuring the energy distribution of the interface states must be developed.

Kelvin probe force spectroscopy (KPFS) or electrostatic force spectroscopy is a technique that enables energy spectroscopy of interface states in the semiconductor bandgap, as described above. Since KPFS does not fix the DC bias voltage but sweeps it over a certain voltage range, it has the advantage of obtaining information on carriers in the energy range corresponding to the swept bias voltage range. For example, the use of electrostatic force spectroscopy to measure the localized energy levels of insulating layers on semiconductor surfaces has been reported to be feasible [22]. Therefore, we can expect that the KPFS method described above can be combined with high–low KPFM to measure the energy distribution of the interface states.

In this study, we propose high-low KPFS using high- and low-frequency AC bias voltages to measure the interface state density inside semiconductors. We derive an analytical expression for the electrostatic force between the tip and the sample that takes into account the charge transfer between the bulk and interface states in the semiconductor. We show that the electrostatic force between the tip and the semiconductor sample strongly depends on the capacitance of the charge depletion region on the surface, and that the analysis of the electrostatic force at low- and high-frequency AC bias voltages can provide information on the interface state density in the semiconductor bandgap. We also demonstrate using a pn-patterned silicon substrate that the interface state density can be measured.

## Theory

**Figure 1:**
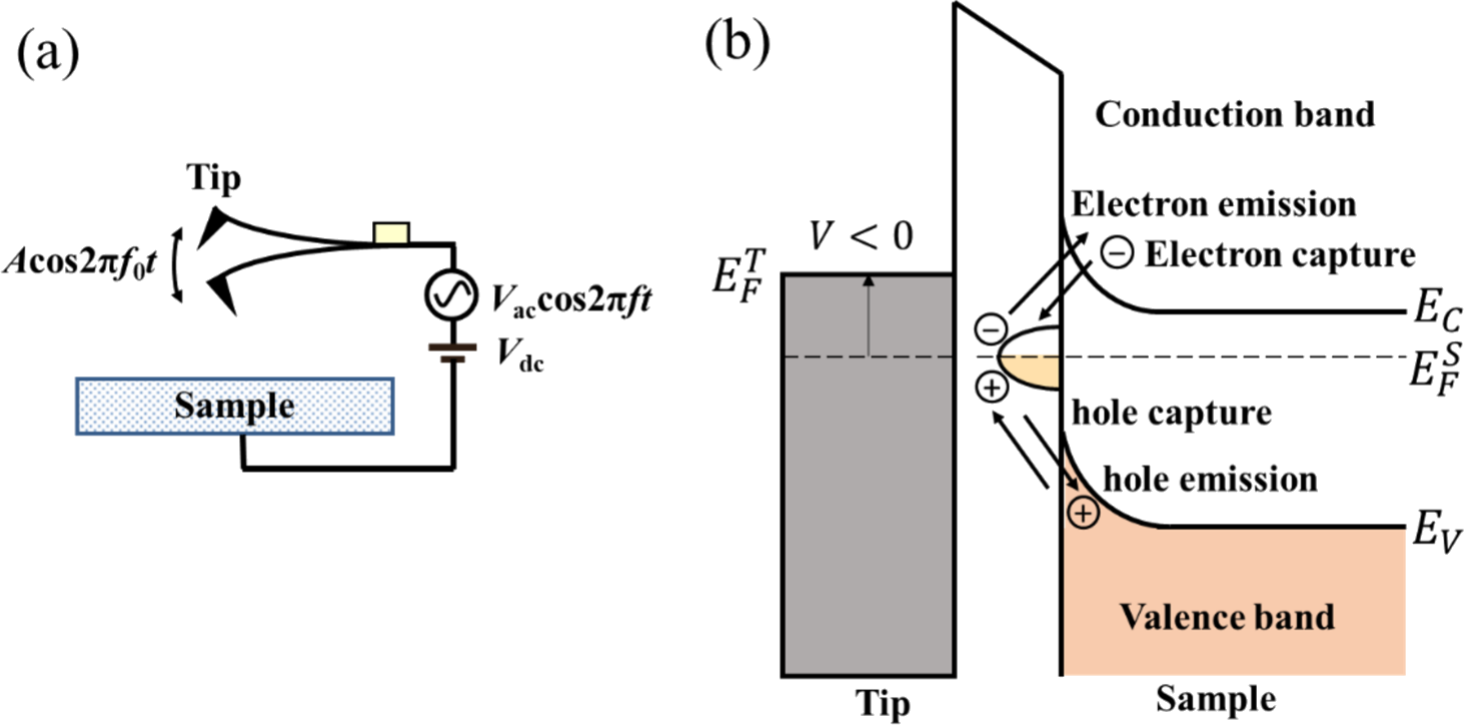
(a) Schematic of the metal tip–gap–semiconductor sample. (b) Energy band diagram of the metal–gap–semiconductor sample. Emission and capture of carriers (electrons and holes) occur between the interface and bulk states of the semiconductor sample when a low-frequency AC bias voltage is applied.

To understand the principle of the high–low KPFS proposed in this study, we discuss the electrostatic forces acting between the tip and the sample when high- and low-frequency AC bias voltages are applied. The tip and the sample are assumed to be metallic and semiconducting, respectively, and a metal–insulator–semiconductor (MIS) structure consisting of the metallic tip, a vacuum gap, and the semiconducting sample is considered (Figure 1a). No oxide film on the semiconductor surface is assumed, and to simplify the discussion, the CPD between the tip and the semiconductor substrate is assumed to be zero.

To investigate the electrostatic force acting between the tip and the semiconductor surface, we use the theoretical model reported by Hudlet and co-workers [23]. For simplicity, let us assume that the tip and the sample are represented by parallel plate capacitors. In this case, the electrostatic force *F*_ele_ acting between the tip and the semiconductor surface is expressed as


[1]

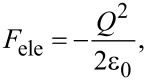



where *Q* is the charge per unit surface area induced on the semiconductor surface and ε_0_ is the dielectric constant of vacuum.

A bias voltage *V*_dc_ + *V*_ac_·cos 2π*ft* is applied between the tip and the semiconductor sample, where *V*_dc_, *V*_ac_, and *f* are the DC bias voltage, amplitude of the AC bias voltage, and modulation frequency of the AC bias voltage, respectively. The modulated electrostatic force *F*_ele_(*f*) between the tip and the surface is expressed as follows using Taylor series expansion:


[2]

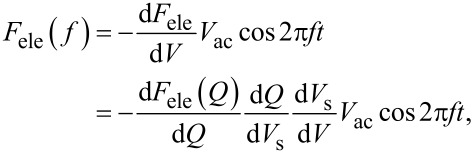



where *V*_s_ is the surface potential of the semiconductor sample.

Next, we consider the charge *Q* induced on the semiconductor surface. When a bias voltage is applied between a metal tip and a semiconductor surface, a surface potential is generated on the semiconductor surface, resulting in, for example, surface charge accumulation, depletion, and inversion states. The relationship between this surface charge and the electrostatic force between the tip and the sample has already been discussed by Hudlet and co-workers [23]. Additionally, there are interface states on the semiconductor surface. Therefore, the contribution of these interface states to the AC component of the electrostatic force must be considered. When an AC bias voltage is applied between the tip and the sample, the bulk Fermi level does not change on the semiconductor side, whereas the interface states move up and down with the conduction and valence bands. This causes capture of carriers (electrons and/or holes) from the bulk side by the interface states and, conversely, emission of carriers from the interface states to the bulk side, which contribute to the electrostatic force (Figure 1b). Therefore, the total charge *Q* induced on the semiconductor surface by the voltage application is given by the sum of the charge *Q*_s_ due to the surface potential *V*_s_ and the charge *Q*_it_ due to the interface states as follows:


[3]

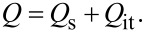



Here, we consider the charge *Q*_s_ due to the surface potential *V*_s_. In the case of an n-type semiconductor, the charge *Q*_s_ as a function of the surface potential *V*_s_ is expressed as follows [23–24]:


[4]

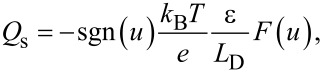




[5]

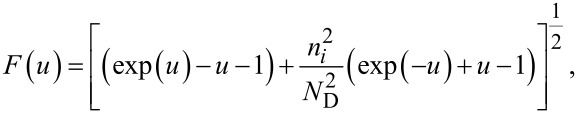




[6]

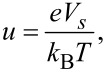




[7]

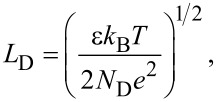



where *N*_D_ is the dopant density, *n*_i_ is the intrinsic carrier density, and ε is the dielectric constant. *L*_D_ is the Debye length for majority carriers (electrons), which characterizes the change in the potential inside the semiconductor. *k*_B_, *T* and *e* are the Boltzmann constant, absolute temperature, and elementary charge (*e* > 0), respectively.

Since the applied voltage *V* is divided between the semiconductor and the gap, the following equation is obtained:


[8]

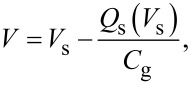



where *C*_g_ is the capacitance per unit surface area due to the gap between the tip and the surface. The charge *Q*_s_ due to the surface potential *V*_s_ is obtained by numerically solving Equations 4–8.

When a positive or negative bias voltage is applied to the MIS structure consisting of the metal tip, gap, and semiconductor sample, three cases exist at the semiconductor surface. For an n-type semiconductor, when a positive bias voltage is applied to the metal tip (*V*_dc_ > 0), electrons (majority carriers) in the semiconductor are attracted to the surface, forming an accumulation layer of electrons. When a small negative bias voltage is applied to the metal tip (*V*_dc_ < 0), electrons (majority carriers) in the semiconductor are depleted from the surface, forming a depletion layer. When a large negative bias voltage is applied to the metal tip, the number of holes (minority carriers) is greater than that of electrons at the surface, and holes are induced in the semiconductor at the surface, forming an inversion layer of holes.

Here, in the accumulation region, since 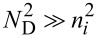 and exp(*u*) ≫ |*u* + 1|, *Q*_s_ is dominated by the first term in Equation 5 and is given by [23–24]


[9]

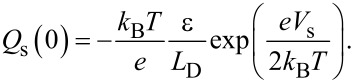



In the depletion region, *Q*_s_ is dominated by the second term −*u* in the square brackets on the right-hand side of Equation 5 and is given by


[10]

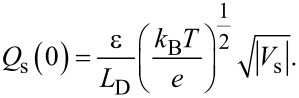



In the strong inversion region, *Q*_s_ is dominated by the fourth term 
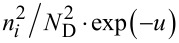
 in the square brackets on the right-hand side of Equation 5 and is given by


[11]

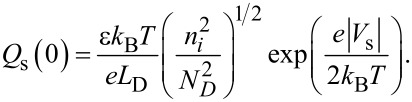



Similar results can be obtained for a p-type semiconductor.

**Figure 2:**
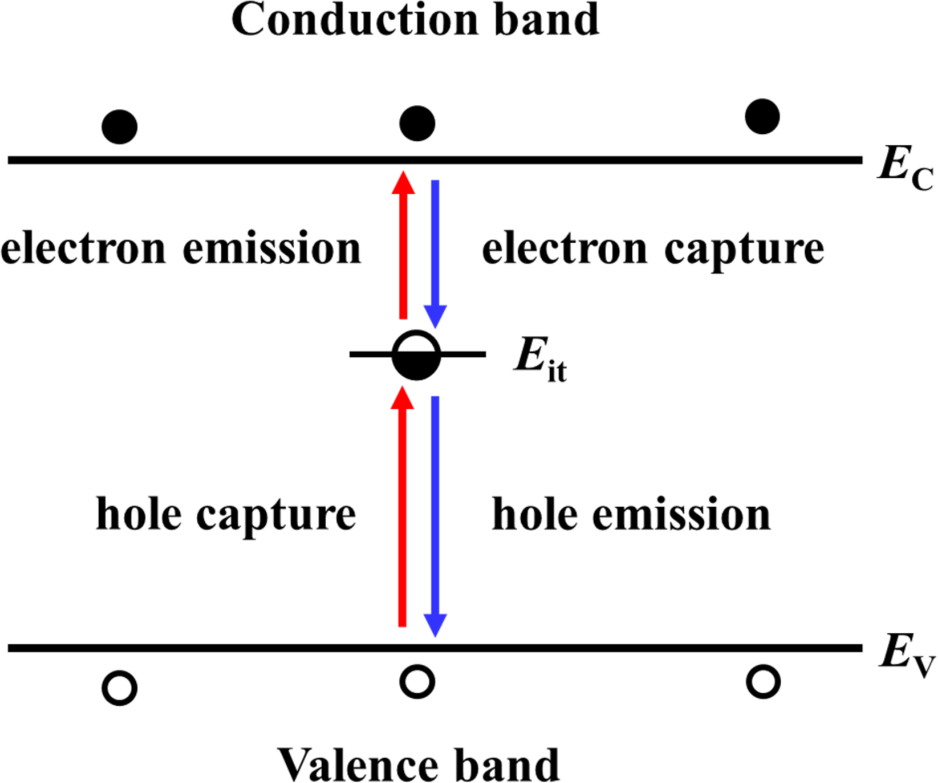
Schematic model of carrier emission and capture between the interface and bulk states of the semiconductor sample.

Next, we consider the charge *Q*_it_ due to the interface states. In indirect-bandgap semiconductors such as Si with a low carrier density below 10^17^ cm^−3^, the charge (electron and hole) transfer between the interface and bulk states at low carrier density can be explained by the model with Shockley–Read–Hall (SRH) statistics [25–26]. This model is based on the charge capture and emission between the interface and bulk states (Figure 2). Assume that 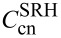 and 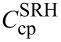 are the capture rates for electrons and holes per electron and hole, respectively, when all interface states are unoccupied, and 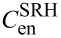 and 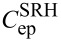 are the emission rates for electrons and holes per electron and hole, respectively. The capture rates per unit volume for electrons and holes (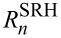 and 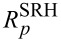) are given by [25–26]


[12]

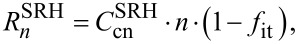




[13]

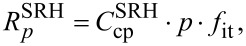



where *f*_it_ is the fraction of occupied interface states. *n* and *p* are the electron and hole densities of the bulk state. Analogously, the emission rates per unit volume for electrons and holes (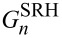 and 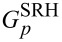) are given by


[14]

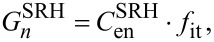




[15]

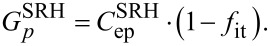



In thermal equilibrium, the amount of capture and the amount of emission for a carrier coincide as follows:


[16]

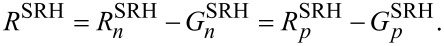



The net generation/recombination rate *R*^SRH^ is given by the following equation [25–26]:


[17]

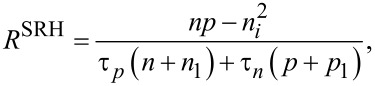



with


[18]

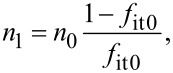




[19]

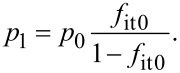



The index 0 indicates equilibrium quantities. For low-level injection, at which the excess minority carrier density is low compared to the equilibrium majority carrier density, the net generation/recombination rate *R*^SRH^ is dominated by the hole (minority carrier) lifetime τ_p_ in n-type semiconductors and the electron (minority carrier) lifetime τ_n_ in p-type semiconductors as follows:


[20]

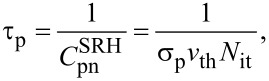




[21]

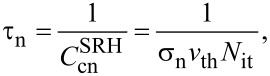



where σ_n_ and σ_p_ are the capture cross sections for electrons and holes, respectively, *v*_th_ is the thermal velocity, and *N*_it_ is the concentration of interface states. These equations indicate that the carrier lifetimes τ_p_ and τ_n_ are reciprocals of the capture rates per single carrier determined by the capture cross sections σ_n_ and σ_p_, thermal velocity *v*_th_, and concentration of interface states *N*_it_, which depend on semiconductor type, temperature, carrier density, and interface state density.

For an n-type Si semiconductor at room temperature, the hole (minority carrier) lifetime τ_p_ as a function of electron (majority carrier) density *n* has been experimentally investigated and is reported to be less than 2.5 × 10^−5^ s for low carrier densities *n* < 5 × 10^17^ cm^−3^ [26]. Additionally, for metal-oxide semiconductor capacitors on Si(100) substrates, the lifetimes τ_n_ and τ_p_ as functions of the sum of the surface potential and the Fermi potential with respect to the midgap have been experimentally investigated [24]. As a result, for Si semiconductors with a low carrier density (small Fermi potential), the lifetime has been reported to be less than 5 × 10^−6^ s. These results indicate that the cutoff frequency *f*_c_ of carrier transport between the interface and bulk states for a Si substrate with a low carrier density is approximately 200 kHz. Therefore, when an AC bias voltage with a frequency higher than this cutoff frequency *f*_c_ is applied between the tip and the Si semiconductor sample, the charge *Q*_it_ caused by the interface states cannot respond to changes in the surface potential. In contrast, when an AC bias voltage with a frequency much lower than *f*_c_ is applied between the tip and the Si semiconductor sample, the charge *Q*_it_ can respond to changes in the surface potential.

### Low KPFS

First, we consider the case in which the frequency of the AC bias voltage is lower than the cutoff frequency *f*_c_ of the carrier transport between the interface and bulk states. The AC bias voltage *V*_ac_·cos 2π*f*_m_*t* is applied between the tip and the surface, where *f*_m_ is the modulation frequency of the AC bias voltage. Since the charge *Q*_it_ due to interface states can follow the change in the surface potential, d*Q*/d*V*_s_ and d*V*_s_/d*V* can be expressed as


[22]

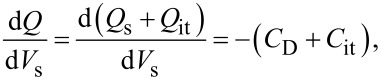




[23]

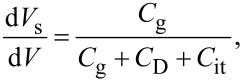



where *C*_D_ and *C*_it_ are the capacitance per unit surface area due to the depletion layer of the semiconductor and the capacitance due to the interface charge, respectively. The applied voltage is divided between the semiconductor and the gap, and the following equation is obtained:


[24]

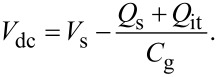



Therefore, the modulation frequency *f*_m_ component of the electrostatic force *F*_ele_(*f*_m_) acting on the probe is expressed as


[25]

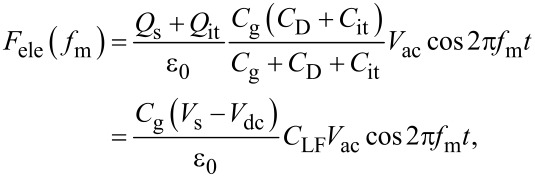




**Figure 3:**
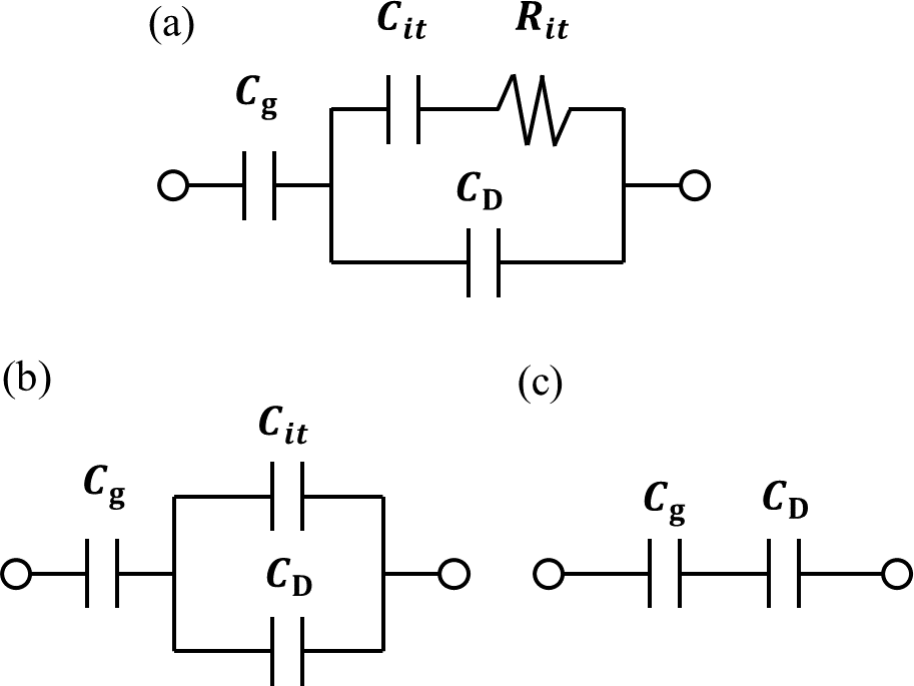
(a) Equivalent circuit of the impedance model (SRH model) of the MIS structure. (b) Equivalent circuit of tip–sample capacitance *C*_LF_ in low-frequency KPFS with a low-frequency AC bias voltage. (c) Equivalent circuit of tip–sample capacitance *C*_HF_ in high-frequency KPFS with a high-frequency AC bias voltage. *C*_g_: capacitance due to the tip–surface gap; *C*_D_: capacitance due to the depletion layer; *C*_it_: capacitance due to interface states; *R*_it_: resistance due to interface states.

[26]

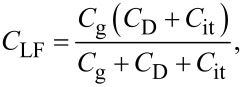



where *C*_LF_ is the low-frequency tip–sample capacitance. The equivalent circuit for this capacitance *C*_LF_ is shown in Figure 3b. Note that this equivalent circuit is equal to the equivalent circuit of the impedance model (SRH model) of the MIS structure (Figure 3a), neglecting the resistance component *R*_it_. This result suggests a similarity between conventional impedance measurements and electrostatic force measurements in semiconductor surface and interface evaluation techniques.

The average distance between the tip and the sample is *z*_to_, and the amplitude and frequency of the vibrating cantilever are *A* and *f*_0_, respectively. The time-varying tip–sample distance is given by


[27]

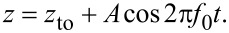



From this equation and the relationship *C*_g_ = ε_0_/*z*, we obtain the following expression:


[28]

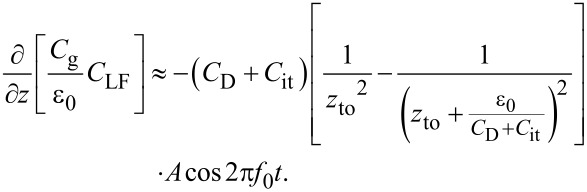



Because of frequency mixing between the electrostatic force due to the AC bias voltage cos 2π*f*_m_*t* and the cantilever vibration cos 2π*f*_0_*t*, *f*_0_ ± *f*_m_ components of the electrostatic force *F*_ele,_*_L_*(*f*_0_ ± *f*_m_) appear:


[29] $$\begin{array}{c} F_{\text{ele},L}\left(f_{0}\pm f_{\text{m}}\right)=-\frac{1}{2}\left(C_{\text{D}}+C_{\text{it}}\right)\left[\frac{1}{z_{\text{to}}{}^{2}}-\frac{1}{{\left(z_{\text{to}}+\frac{\varepsilon_{0}}{C_{\text{D}}+C_{\text{it}}}\right)}^{2}}\right]\\ \cdot \left(V_{\text{s}}-V_{\text{dc}}\right)V_{\text{ac}}A\cos2\pi \left(f_{0}\pm f_{\text{m}}\right)t. \end{array}$$

When the electrostatic force is detected by the FM method, the electrostatic force *F*_ele,_*_L_*(*f*_0_ ± *f*_m_) is demodulated into the *f*_m_ component of the frequency shift Δ*f_L_*(*f*_m_), which is expressed as


[30]

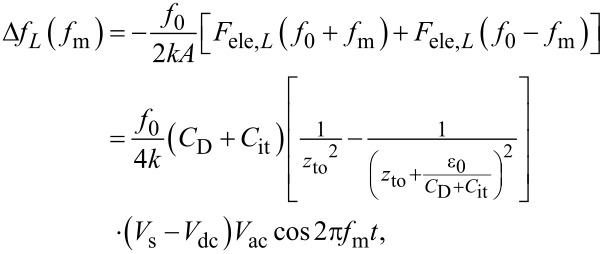



where *k* is the spring constant of the cantilever. This equation indicates that the slope of the dependence of the *f*_m_ component of the frequency shift Δ*f_L_*(*f*_m_) on the DC bias voltage *V*_dc_ (Δ*f_L_*(*f*_m_)–*V*_dc_ curve) is proportional to the capacitance inside the semiconductor at a low-frequency AC bias (*C*_D_ + *C*_it_).

### High KPFS

Next, we consider the case in which the frequency of the AC bias voltage is higher than the cutoff frequency *f*_c_ of the carrier transport between the interface and bulk states. We assume that the heterodyne FM method [21] is used and that an AC bias voltage with a high frequency near twice the vibration frequency of the cantilever *V*_ac_·cos 2π(2*f*_0_ + *f*_m_)*t* is applied (that is, *f* = 2*f*_0_ + *f*_m_). In the high-frequency case, the contribution from the interface charge *Q*_it_ due to interface states cannot follow the change in surface potential *V*_s_, so this contribution can be neglected. Therefore, d*Q*/d*V*_s_ and d*V*_s_/ d*V* can be expressed as


[31]

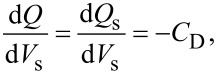




[32]

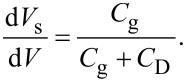



Since the applied voltage is divided between the semiconductor and the gap, the following equation is obtained:


[33]

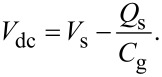



Therefore, the modulation frequency (2*f*_0_ + *f*_m_) component of the electrostatic force *F*_ele_(2*f*_0_ + *f*_m_) acting on the tip is expressed as


[34]

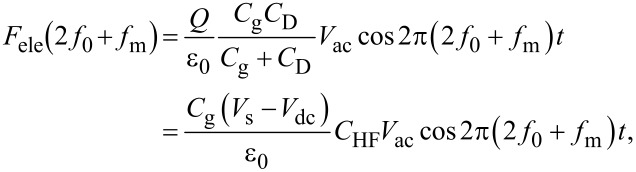




[35]

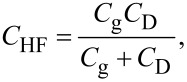



where *C*_HF_ is the high-frequency tip–sample capacitance. The equivalent circuit for this capacitance *C*_HF_ is shown in Figure 3c. Note that this equivalent circuit is equal to the equivalent circuit of the impedance of the MIS structure derived from the SRH model (Figure 3a), neglecting the capacitance component *C*_it_ and resistance component *R*_it_ due to the semiconductor interface states.

Now, the capacitance *C*_D_ of the depletion layer of the MIS structure is given by *C*_D_ = |d*Q*_s_/d*V*_s_|. In the charge accumulation region, from Equation 9, *C*_D_ is given by


[36]

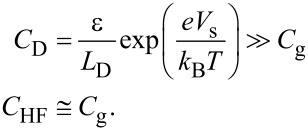



In the depletion region, from Equation 10, *C*_D_ is given by


[37]

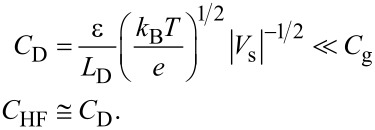



In the inversion region, from Equation 11, *C*_D_ is given by


[38]

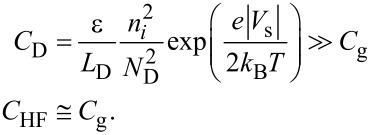



Therefore, in the depletion region, we can obtain the following expression:


[39]

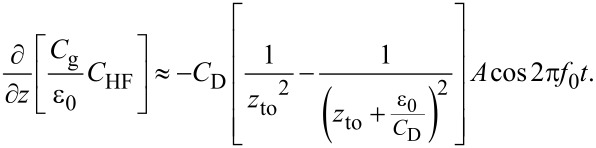



In the accumulation and inversion regions, we can obtain the following expression:


[40]

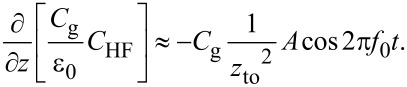



Because of frequency mixing between the electrostatic force due to the AC bias voltage cos 2π(2*f*_0_ + *f*_m_)*t* and the cantilever vibration cos2π*f*_0_*t*, the *f*_0_ + *f*_m_ component of the electrostatic force *F*_ele,_*_H_*(*f*_0_ + *f*_m_) appears:

- In the depletion region,

[41]

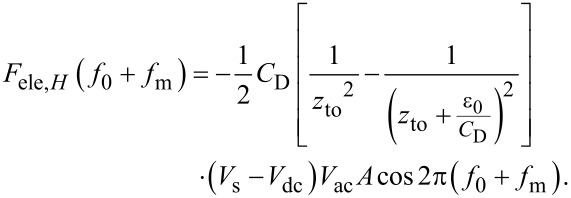

- In the accumulation and inversion regions,

[42] $$F_{\text{ele},H}\left(f_{0}+f_{\text{m}}\right)=-\frac{1}{2}C_{\text{g}}\frac{1}{z_{\text{to}}{}^{2}}\left(V_{\text{s}}-V_{\text{dc}}\right)V_{\text{ac}}A\cos2\pi \left(f_{0}+f_{\text{m}}\right).$$

In the FM method, the electrostatic force *F*_ele,_*_H_*(*f*_0_ + *f*_m_) is demodulated into the *f*_m_ component of the frequency shift Δ*f_H_*(*f*_m_). The resulting *f*_m_ component of the frequency shift Δ*f_H_*(*f*_m_) is expressed in the depletion region as


[43]

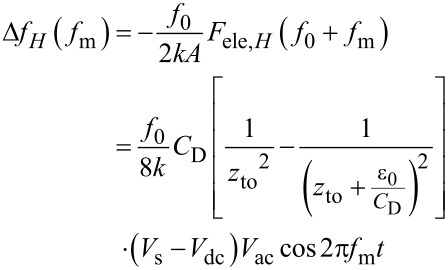



and in the accumulation and inversion regions as


[44]

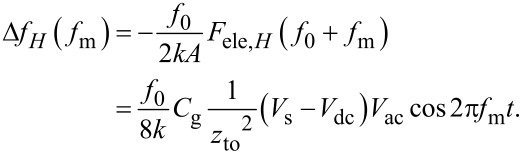



In the above equations, the slope of the dependence of the *f*_m_ component of the frequency shift Δ*f_H_*(*f*_m_) on *V*_dc_ (Δ*f_H_*(*f*_m_)–*V*_dc_ curve) is proportional to the capacitance *C*_D_ inside the semiconductor in the depletion region and to the gap capacitance *C*_g_ in the accumulation and inversion regions. Note that at a high-frequency AC bias voltage, as shown in Equation 43 and Equation 44, only the *f*_0_ + *f*_m_ component of the electrostatic force *F*_ele,_*_H_*(*f*_0_ + *f*_m_) is demodulated, while at a low-frequency AC bias voltage, as shown in Equation 30, the *f*_0_ + *f*_m_ and *f*_0_ − *f*_m_ components of the electrostatic force *F*_ele,_*_L_*(*f*_0_ ± *f*_m_) are demodulated. Therefore, the coefficients in Equation 43 and Equation 44 are 1/2 of those in Equation 30.

### High–low KPFM

Here, we consider the derivation of the surface potential *V*_s_ induced by the interface states. When the KPFM measurement is performed using a low-frequency AC bias voltage, the DC bias voltage that makes the modulation component of the frequency shift Δ*f_L_*(*f*_m_) zero is


[45]

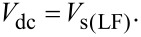



Here, *V*_s(LF)_ can be thought of as reflecting information about the sum of the surface potential (band bending) due to the interface states and the CPD between the metal tip and the bulk state of the sample. This is because when a low-frequency AC bias voltage is used, the charge *Q*_it_ can respond to changes in the surface potential. In contrast, when the KPFM measurement is performed using a high-frequency AC bias voltage, the DC bias voltage that makes the modulation component of the frequency shift Δ*f_H_*(*f*_m_) zero is


[46]

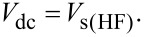



Here, *V*_s(HF)_ can be thought of as reflecting information about the CPD between the metal tip and the bulk state of the sample. This is because when a high-frequency AC bias voltage is used, the charge *Q*_it_ caused by the interface states cannot respond to changes in the surface potential. Therefore, as shown in the next equation, high–low KPFM, which measures the difference between *V*_s(LF)_ with a low-frequency AC bias voltage and *V*_s(HF)_ with a high-frequency AC bias voltage, reflects the information of the surface potential (band bending) due to the interface states [21–22].


[47]

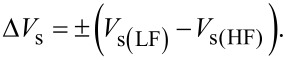



Regarding the selection of ±, + is for applying a bias voltage to the sample and − is for applying a bias voltage to the tip. The + and − signs of Δ*V*_s_ indicate upward and downward band bending at the interface, respectively.

### High–low KPFS

Next, let us consider the derivation of the interface state density *D*_it_ from Equation 30 and Equation 43, which relate the modulation components of the frequency shift to the AC bias voltage at low and high frequencies. In the case of a low-frequency AC bias, from Equation 30, the slope of the Δ*f_L_*(*f*_m_)–*V*_dc_ curve is proportional to the low-frequency capacitance inside the semiconductor (*C*_D_ + *C*_it_). In the case of a high-frequency AC bias, from Equation 43, twice the slope of the Δ*f_H_*(*f*_m_)–*V*_dc_ curve in the depletion region is proportional to the high-frequency capacitance within the semiconductor (*C*_D_ ). Using these relationships, the interface state density *D*_it_ at each DC bias voltage *V*_dc_ can be obtained by taking the difference in the slope of the dependence of Δ*f*(*f*_m_) on the DC bias voltage for low and high frequencies (the capacitance *C*_it_ due to the interface charge) [24] as follows:


[48]

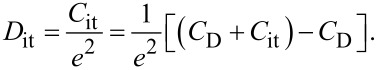



### Δ*f*–*V*_dc_ curve with low- and high-frequency AC bias voltages

We consider the derivation of the Δ*f*–*V*_dc_ curve. The DC electrostatic force *F*_ele_(0) between the tip and the surface when using an AC bias voltage with frequency *f* (= *f*_m_ or 2*f*_0_ + *f*_m_) is expressed as follows using Taylor series expansion up to the second-order terms:


[49]

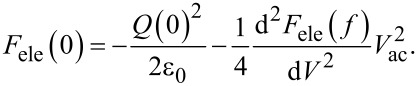



The contribution of the second-order terms of the Taylor series expansion of the electrostatic force is much smaller than that of the zeroth-order term and can be ignored. *Q*_s_ can be obtained by solving Equations 4–8 numerically, but the relationship between the DC bias voltage *V*_dc_ and the frequency shift Δ*f* cannot be understood analytically.

Therefore, assuming that the vibration amplitude of the cantilever is very small compared to the length of the electrostatic force interaction region, we can obtain the analytical relationship between *V*_dc_ and the frequency shift Δ*f*. The gradient of the electrostatic force is given by


[50]

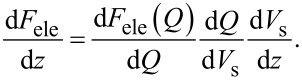



From Equations 24 and 33 and the relationship *C*_g_ = ε_0_/*z*, d*V*_s_/d*z* is given by


[51]

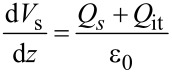



and


[52]

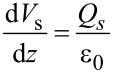



for low- and high-frequency AC bias voltages, respectively. From Equations 22, 24, 31, and 33, the frequency shift of the electrostatic force is given by


[53]

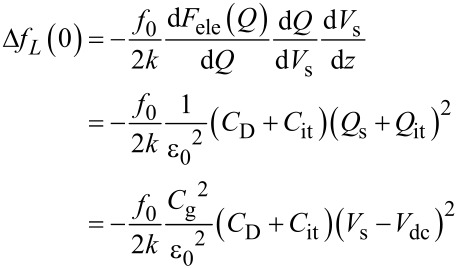



and


[54]

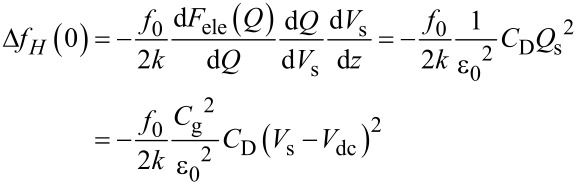



for low- and high-frequency AC bias voltages, respectively. As shown in Equations 36–38, *C*_D_ in the charge depletion region is very different from *C*_D_ in the charge accumulation and charge inversion regions. Thus, these equations suggest that the Δ*f_L_*–*V*_dc_ curve for a low-frequency AC bias voltage is almost parabolic with respect to *V*_dc_ when *C*_D_ < *C*_it_, while the Δ*f_H_*–*V*_dc_ curve for a high-frequency AC bias voltage is divided into three regions with respect to *V*_dc_. These analytical equations do not necessarily quantitatively agree with the experimental results because they approximate small cantilever vibration amplitudes, but they can qualitatively explain the behavior of Δ*f*–*V*_dc_ curves.

## Experimental

**Figure 4:**
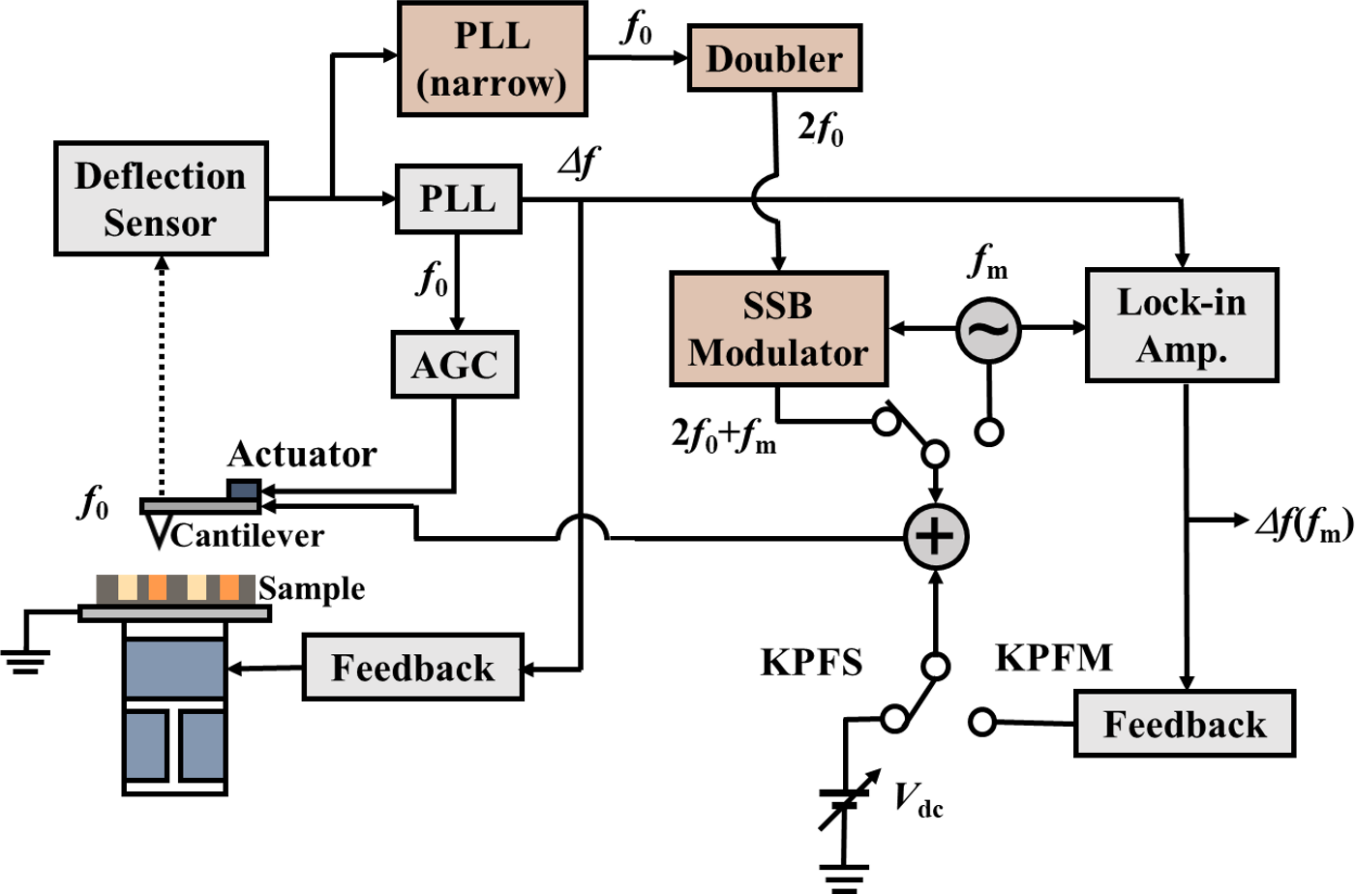
Block diagram of AFM and high–low KPFS. In low KPFS, a signal *V*_ac_·cos 2π*f*_m_*t* is generated by the oscillator. In high KPFS, a signal cos 2π(2*f*_0_)*t* of twice the resonance frequency synchronized with the cantilever oscillation is generated by the PLL and the doubler, and a signal *V*_ac_·cos 2π(2*f*_0_ + *f*_m_)*t* is generated by mixing the signal cos 2π(2*f*_0_)*t* and the signal cos 2π*f*_m_*t* in the SSB modulator. The figure was adapted from [20] (© 2020 Y. Sugawara et al., published by IOP Publishing Ltd., distributed under the terms of the Creative Commons Attribution 4.0 International License, https://creativecommons.org/licenses/by/4.0)

Figure 4 shows the block diagram of AFM and high–low KPFS using AC bias voltages with high and low frequencies. The FM method was used to detect the tip–sample interaction force. The cantilever displacement signal measured using the displacement detection system was controlled by an automatic gain control (AGC) circuit to keep the cantilever vibration amplitude *A* constant, and the frequency shift Δ*f* of the cantilever was measured using a phase-locked loop (PLL) circuit (SPECS GmbH: Nanonis OC4). AFM measurements were performed by controlling the tip–sample distance so that the frequency shift of the cantilever (Δ*f*_set_) was constant. In the low KPFS measurement using a low-frequency AC bias, the AC bias voltage *V*_ac_·cos 2π*f*_m_*t* was generated by an oscillator. In contrast, in the high KPFS measurement using a high-frequency AC bias voltage, the signal cos 2π(2*f*_0_)*t*, which is a signal of twice the frequency synchronized with the cantilever vibration signal cos 2π*f*_0_*t*, was generated by a PLL circuit with a narrow bandwidth and a doubler (amplitude modulator for second-harmonic generation) (Zurich Instruments: HF2LI-PLL and HF2LI-MOD). By mixing this signal and the signal from the oscillator cos 2π*f*_m_*t* in a single-sideband (SSB) modulator (Zurich Instruments: HF2LI-MOD), the AC bias signal *V*_ac_·cos 2π(2*f*_0_ + *f*_m_)*t* was generated. The bias voltage, which is the sum of the DC bias voltage *V*_dc_ and the AC bias voltage (*V*_ac_·cos 2π*f*_m_*t* or *V*_ac_·cos 2π(2*f*_0_ + *f*_m_)*t*), was applied to the tip, and the ground was connected to the sample. The modulation component of the frequency shift of the cantilever Δ*f*(*f*_m_) was detected using a lock-in amplifier (Zurich Instruments: HF2LI). Bias spectral data were obtained by measuring Δ*f*(*f*_m_) and Δ*f* as a function of the DC bias voltage *V*_dc_ while keeping the distance between the tip and the sample constant. These experiments were performed in a vacuum environment using a JEOL scanning probe microscope (JEOL: JSPM-4210).

**Figure 5:**
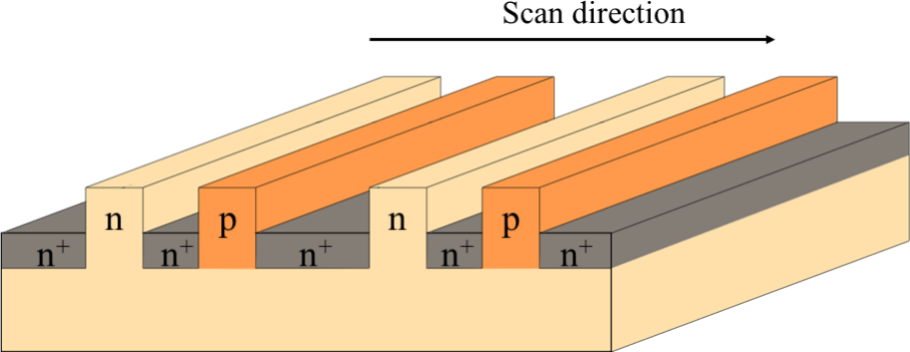
Schematic of the silicon substrate with three types of impurity pattern, that is, n-, p-, and, n^+^-type regions, used for the measurement.

A silicon substrate patterned with n- and p-type impurities was used as a semiconductor sample [22,27–28]. Figure 5 shows the dopant pattern of the silicon substrate used in the measurements and the impurity concentrations. As shown in Figure 5, the silicon substrate has three types of impurity regions: an n-type region, a p-type region, and an n^+^-type region. The impurity concentrations of the n, p, and n^+^ regions are 1 × 10^15^ cm^−3^, 2 × 10^16^ cm^−3^, and 5 × 10^19^ cm^−3^, respectively.

As a force sensor, a PtIr-coated conductive cantilever (NanoWorld: NCHPt) was used. The resonance frequency *f*_0_, force constant *k*, and *Q* of the PtIr-coated cantilever were 292.68 kHz, 42 N/m, and 8406, respectively.

The cantilever oscillation amplitude for the high–low KPFS measurement was set to *A* = 10 nm throughout the experiment. The AC bias voltage was *V*_ac_ = 0.5 V, and the modulation frequencies of the AC bias voltage in high KPFS and low KPFS were 2*f*_0_ + *f*_m_ = 542.3 kHz and *f*_m_ = 100 Hz, respectively.

## Results and Discussion

**Figure 6:**
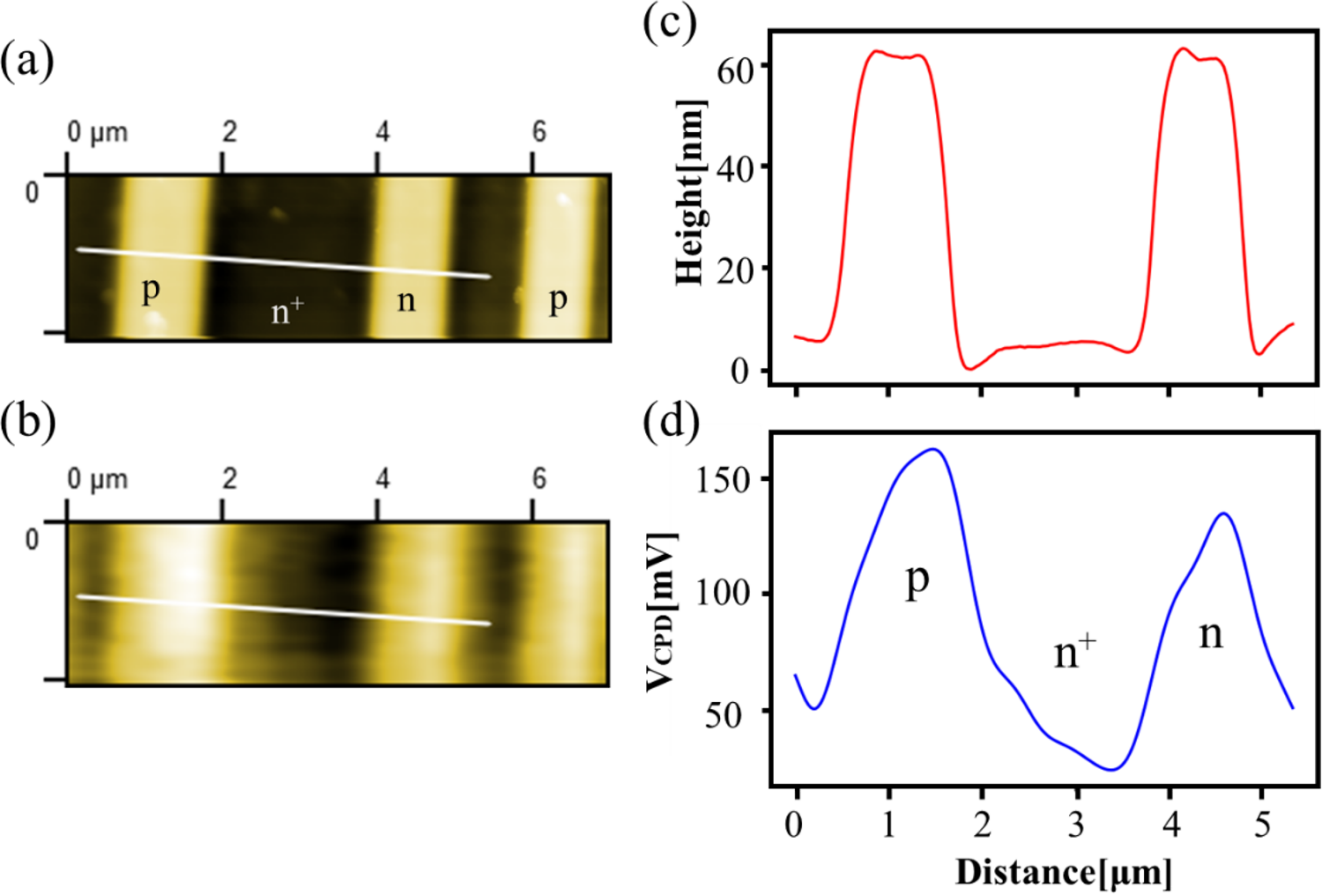
(a) Surface topography and (b) CPD image of the pn-patterned Si surface. The CPD image was obtained by KPFM using an AC bias voltage with a low modulation frequency of *f*_m_ = 100 Hz. The scan size was 7 μm × 2.1 μm. (c) Line profile corresponding to the white line in panel (a); (d) line profile corresponding to the white line in panel (b).

First, we performed AFM/KPFM measurements on the pn-patterned Si surface to identify the dopant regions on the semiconductor surface. Figure 6a and Figure 6b show the topographic and CPD images of the pn-patterned Si substrate, respectively. The CPD image in Figure 6b was obtained by KPFM using an AC bias voltage with a low modulation frequency of *f*_m_ = 100 Hz. Figure 6c and Figure 6d show the line profiles corresponding to the white lines in Figure 6a and Figure 6b, respectively. From the topographic image and the line profile, there are rodlike protrusions on the surface with a width of approximately 1 μm and a height of approximately 60 nm. Compared with the data of the pn-patterned Si substrate in Figure 5, these rodlike protrusions correspond to p-type or n-type regions, and the low areas between the rodlike protrusions correspond to n^+^-type regions. From the CPD image and the line profile, there is a difference of approximately 30–40 mV between the CPD values of the two rodlike protrusions; the region with the highest CPD value is the p-type region, the region with a CPD value 30–40 mV below the highest value is the n-type region, and the region with the lowest CPD value is the n^+^-type region. From these CPD values, the n^+^-, n-, and p-type regions can be assigned as shown in Figure 6d [22,27–28].

**Figure 7:**
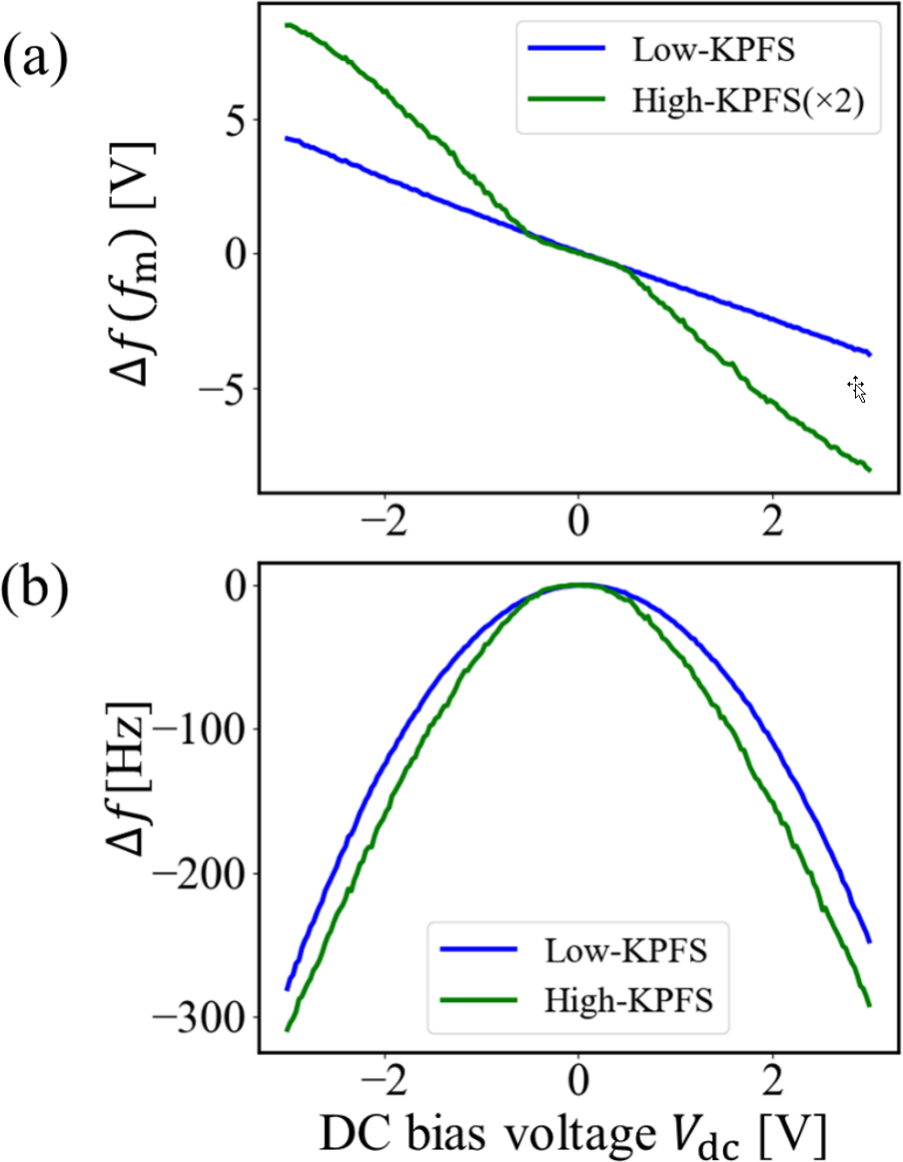
(a) Δ*f*(*f*_m_)–*V*_dc_ curves and (b) Δ*f*–*V*_dc_ curves obtained on the n-type Si surface. The tip–sample distance was set with Δ*f*_set_ = −300 Hz (*z*_to_ ≈ 15 nm) when *V*_dc_ = 3 V and *V*_ac_ = 0.5 V were applied between the tip and the sample.

Next, we performed high–low KPFS experiments at the center point in the n-type region shown in Figure 6a. Figure 7a shows the dependence of the modulation component of the frequency shift Δ*f*(*f*_m_) on the DC bias voltage *V*_dc_ in high–low KPFS (Δ*f*(*f*_m_)–*V*_dc_ curves). Since the coefficients in Equations 43 and 44 for high KPFS are 1/2 compared with those in Equation 30 for low KPFS, the modulation component of the frequency shift Δ*f*(*f*_m_) in high KPFS is doubled. The tip–sample distance was fixed at Δ*f*_set_ = −300 Hz (*z*_to_ ≈ 15 nm) as the frequency shift set point when the DC bias voltage *V*_dc_ = 3 V and the AC bias voltage with amplitude *V*_ac_ = 0.5 V were applied. In low KPFS, the Δ*f*(*f*_m_)–*V*_dc_ curve shows an almost linear behavior with respect to *V*_dc_ from −3.5 V to +3.5 V. In contrast, in high KPFS, the Δ*f*(*f*_m_)–*V*_dc_ curve does not show a linear behavior with respect to *V*_dc_ and is roughly divided into three regions: (i) −3.5 V to −0.5 V, (ii) −0.5 V to +0.5 V, and (iii) +0.5 V to +3.5 V. The slope of the curve is larger in regions (i) and (iii) and smaller in region (ii). These experimental results on the Δ*f*(*f*_m_)–*V*_dc_ curves in Figure 7a agree well with the theoretical expectation that the Δ*f_L_*(*f*_m_)–*V*_dc_ curve for low-frequency AC bias voltages is nearly linear with respect to *V*_dc_ with slope *C*_D_ + *C*_it_ and that the Δ*f_H_*(*f*_m_)–*V*_dc_ curve for high-frequency AC bias voltages is divided into three regions with respect to *V*_dc_ with slopes of *C*_D_ in the charge depletion region and of *C*_g_ in the charge accumulation and charge inversion regions. A similar result that the Δ*f*(*f*_m_)–*V*_dc_ curve is divided into three regions has already been observed in organic semiconductor samples by Schumacher and co-workers [29].

The DC bias voltages that reduced Δ*f*(*f*_m_) to zero are slightly different depending on the frequency of the AC bias voltage: *V*_s(LF)_ ≈ 47.5 mV and *V*_s(HF)_ ≈ 12.0 mV for low- and high-frequency AC bias voltages, respectively. The difference between *V*_s(LF)_ and *V*_s(HF)_ is Δ*V*_s_ = −35.5 mV, which indicates that the interface band is bent downward in the n-type region, suggesting the presence of donor-like interface states [22].

Figure 7b shows the dependence of the frequency shift Δ*f* on DC bias voltage *V*_dc_ in high–low KPFS (Δ*f*–*V*_dc_ curves) simultaneously measured with Figure 7a. The Δ*f*–*V*_dc_ curve is almost parabolic for the low-frequency AC bias voltage. In contrast, the Δ*f*–*V*_dc_ curve for the high-frequency AC bias voltage is highly distorted from parabolic, especially in the *V*_dc_ range of −3.5 V to −0.5 V (the charge accumulation region) and +0.5 V to +3.5 V (the charge inversion region). These experimental results on the Δ*f*–*V*_dc_ curves in Figure 7b also agree well with the theoretical expectation that the Δ*f*–*V*_dc_ curve is almost parabolic for low-frequency AC bias voltages, while it is divided into three regions with respect to *V*_dc_ for high-frequency AC bias voltages.

This is the first observation of the phenomenon in which the Δ*f*(*f*_m_)–*V*_dc_ and Δ*f*–*V*_dc_ curves are highly dependent on the frequency of the applied AC bias voltage. These results experimentally show that carrier transport between the bulk and interface states of a semiconductor sample is strongly affected by the frequency of the AC bias voltage and that there is indeed a difference between the low-frequency tip–sample capacitance (*C*_LF_) and the high-frequency tip–sample capacitance (*C*_HF_).

**Figure 8:**
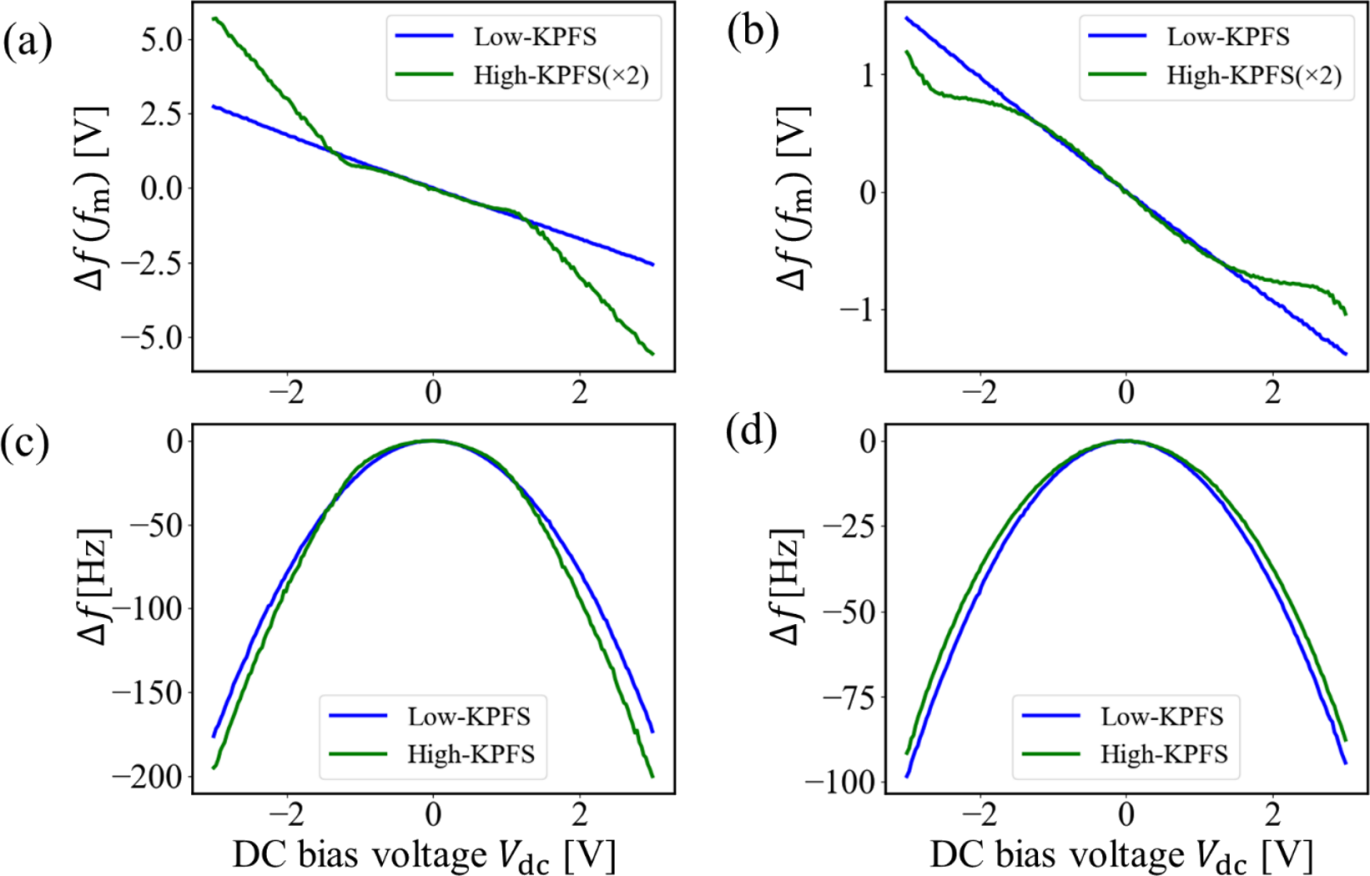
Δ*f*(*f*_m_)–*V*_dc_ curves obtained on the n-type Si surface at (a) Δ*f*_set_ = −200 Hz and (b) Δ*f*_set_ = −100 Hz. Δ*f*–*V*_dc_ curves obtained on the n-type Si surface at (c) Δ*f*_set_ = −200 Hz and (d) Δ*f*_set_ = −100 Hz.

Next, we performed high–low KPFS measurements at the same measurement points as in Figure 7, varying the tip–sample distance. Figure 8a and Figure 8b show the measured Δ*f*(*f*_m_)–*V*_dc_ curves when the tip–sample distance was fixed with Δ*f*_set_ = −200 Hz (*z*_to_ ≈ 23 nm) and Δ*f*_set_ = −100 Hz (*z*_to_ ≈ 40 nm), respectively, as the frequency shift set point. The DC bias voltage *V*_dc_ = 3 V and the AC bias voltage *V*_ac_ = 0.5 V were the same as those used in the case of Figure 7. The modulation component of the frequency shift Δ*f*(*f*_m_) in high KPFS was doubled. Similarly, Figure 8c and Figure 8d show the Δ*f*–*V*_dc_ curves at Δ*f*_set_ = −200 Hz (*z*_to_ ≈ 23 nm) and Δ*f*_set_ = −100 Hz (*z*_to_ ≈ 40 nm) simultaneously measured with Figure 8a and Figure 8b, respectively.

The Δ*f*(*f*_m_)–*V*_dc_ curves in low KPFS show an almost linear behavior at both tip–sample distances. In contrast, the Δ*f*(*f*_m_)–*V*_dc_ curves in high KPFS do not show a linear behavior and are roughly divided into three regions. The Δ*f*(*f*_m_)–*V*_dc_ curves in high KPFS show that the DC bias voltage region (ii) corresponding to the charge depletion region becomes wider from −1.5 V to +1.5 V (Figure 8a) and from −3.3 V to +3.3 V (Figure 8b) as the tip–sample distance increases. The wider voltage region (ii) is due to the smaller ratio of the DC bias voltage divided inside the semiconductor. Furthermore, as the tip–sample distance increases, the Δ*f*–*V*_dc_ curves become nearly parabolic not only for low-frequency AC bias voltages but also for high-frequency AC bias voltages. The reason why the Δ*f*–*V*_dc_ curve for high-frequency AC bias voltages becomes almost parabolic as the tip–sample distance increases is because the range of the DC bias voltage corresponding to the charge depletion region is extended.

**Figure 9:**
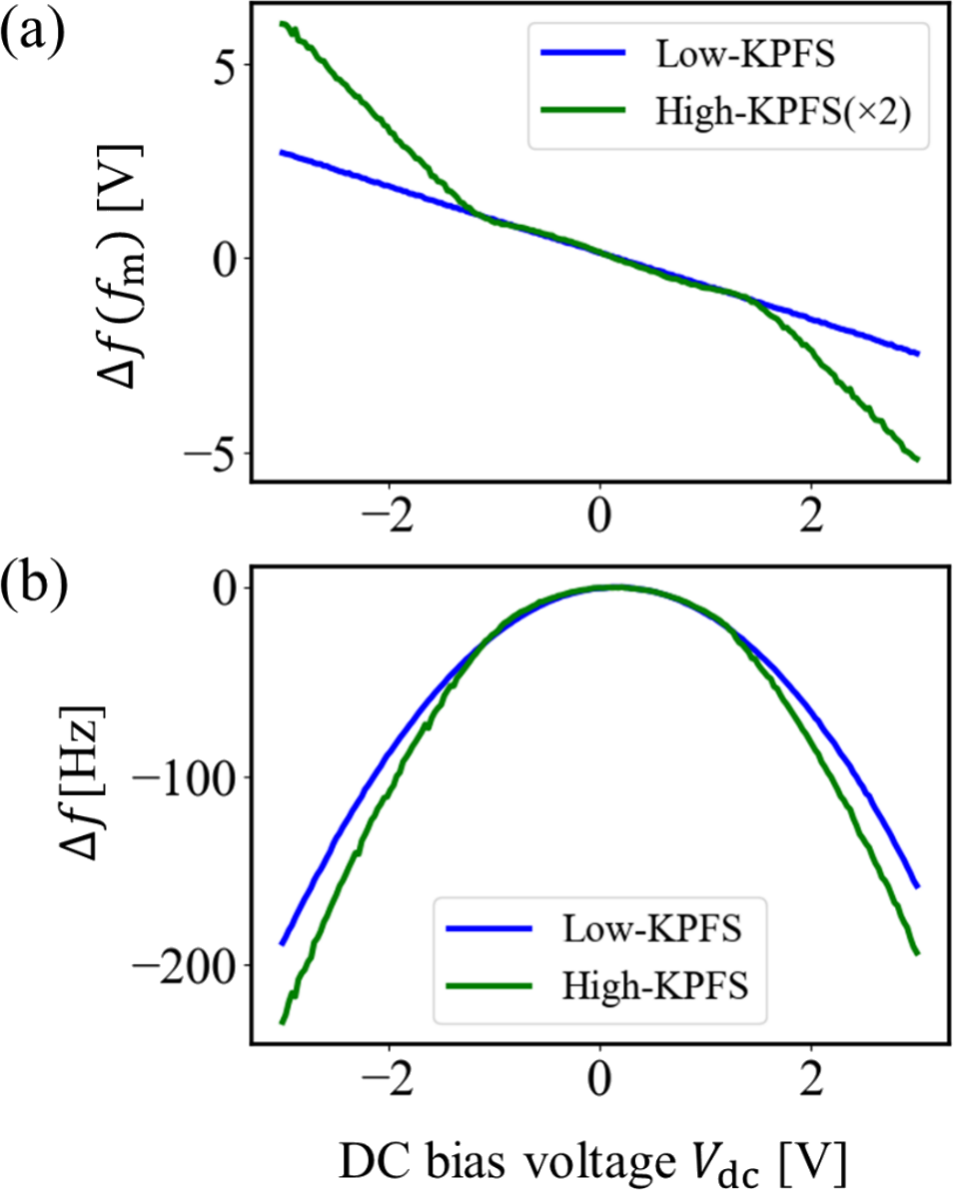
(a) Δ*f*(*f*_m_)–*V*_dc_ curves and (b) Δ*f*–*V*_dc_ curves obtained on the p-type Si surface. The tip–sample distance was set with Δ*f*_set_ = −200 Hz (*z*_to_ ≈ 23 nm) when *V*_dc_ = 3 V and *V*_ac_ = 0.5 V were applied between the tip and the sample.

We also performed high–low KPFS measurements at the center point in the p-type region in Figure 6a. Figure 9a and Figure 9b show the measured Δ*f*(*f*_m_)–*V*_dc_ curves and Δ*f*–*V*_dc_ curves, respectively. The tip–sample distance was fixed with Δ*f*_set_ = −200 Hz (*z*_to_ ≈ 23 nm) as the frequency shift set point when the DC bias voltage *V*_dc_ = 3 V and the AC bias voltage with amplitude *V*_ac_ = 0.5 V were applied. In Figure 9a, the Δ*f*(*f*_m_)–*V*_dc_ curve in low KPFS shows an almost linear behavior from −3.5 V to +3.5 V, while the Δ*f*(*f*_m_)–*V*_dc_ curve in high KPFS is roughly divided into three regions: (i) −3.5 V to −1.5 V, (ii) −1.5 V to +1.5 V, and (iii) +1.5 V to +3.5 V. The slope of the curve is larger in regions (i) and (iii) and smaller in region (ii). The DC bias voltages that reduced Δ*f*(*f*_m_) to zero were estimated to be *V*_s(LF)_ ≈ 169.4 mV and *V*_s(HF)_ ≈ 143.3 mV for low- and high-frequency AC bias voltages, respectively. The difference between *V*_s(LF)_ and *V*_s(HF)_ is Δ*V*_s_ = −25.1 mV. This indicates that the interface band is bent downward in the p-type region, consistent with the previous high–low KPFM results [22].

Furthermore, in Figure 9b, the Δ*f*–*V*_dc_ curve is almost parabolic for the low-frequency AC bias voltage, whereas it is highly distorted from parabolic for the high-frequency AC bias voltage. Thus, the Δ*f*(*f*_m_)–*V*_dc_ and Δ*f*–*V*_dc_ curves measured in the p-type region are highly dependent on the frequency of the applied AC bias voltage, which is in good agreement with those measured in the n-type region as well as the theoretical prediction.

The dependence of the Δ*f*(*f*_m_)–*V*_dc_ and Δ*f*–*V*_dc_ curves on the frequency of the AC bias voltage is due to the dependence on the contribution of the capacitance *C*_it_ caused by the interface states to the electrostatic force. In other words, when a low-frequency AC bias is used, the capacitance inside the semiconductor is given by the sum of the depletion layer capacitance *C*_D_ and the capacitance *C*_it_ due to the interface states according to Equation 30. In contrast, when a high-frequency AC bias is used, the depletion layer capacitance *C*_D_ or the gap capacitance *C*_g_ has a significant effect on the electrostatic force because the capacitance *C*_it_ due to the interface states makes no contribution to the electrostatic force according to Equations 43 and 44.

Finally, we estimated the interface state density *D*_it_ in the n-type region on the pn-patterned Si surface using the measured Δ*f*(*f*_m_)–*V*_dc_ curve in Figure 7a for the closest distance between the tip and the surface. In depletion region (ii), the slope of the Δ*f*(*f*_m_)–*V*_dc_ curve in low KPFS is slightly larger than that in high KPFS. From Equations 30 and 43, at *V*_dc_ = −0.3 V, the capacitances per unit area inside the semiconductor were estimated to be *C*_D_ + *C*_it_ ≃ 3.41 × 10^−23^ F/cm^2^ in low KPFS and *C*_D_ ≃ 2.94 × 10^−23^ F/cm^2^ in high KPFS. The parameters used in the estimations of the interface state density were the same as those used in the experiments (*f*_0_ = 292.68 kHz, *k* = 42 N/m, and *V*_ac_ = 0.5 V). The average distance between the tip and the surface was assumed to be *z*_t0_ ≃ 15 nm. Hence, the capacitance per unit area due to the surface states was estimated to be *C*_it_ ≃ 4.67 × 10^−24^ F/cm^2^. From Equation 48, the interface state density was calculated to be *D*_it_ ≃ 1.82 × 10^14^ cm^−2^ eV at *V*_dc_ = −0.3 V. This value is reasonable for the interface state density on the pn-patterned Si surface. This is the first estimate of the interface state density using the high–low KPFS method and demonstrates the usefulness of high–low KPFS.

## Conclusion

In this study, we proposed high-low KPFS using high-frequency and low-frequency AC bias voltages to measure the interface state density in semiconductors. We derived an analytical expression for the electrostatic force between the tip and the sample that takes into account the charge transfer between the bulk and interface states in the semiconductor. From the analytical equation, we found that the slopes of the Δ*f*(*f*_m_)–*V*_dc_ curves for low- and high-frequency AC bias voltages depend on the capacitances *C*_D_ + *C*_it_ and *C*_D_ between the tip and the sample, respectively. We also showed that the analysis of the difference between *C*_D_ + *C*_it_ and *C*_D_ for low- and high-frequency AC bias voltages provides information on the interface state density *D*_it_ in the semiconductor bandgap.

Experimentally, Δ*f*(*f*_m_)–*V*_dc_ and Δ*f*–*V*_dc_ curves were measured for impurity-doped Si samples (n- and p-types). When a low-frequency AC bias voltage was used, the Δ*f*(*f*_m_)–*V*_dc_ curves were almost linear, and the Δ*f*–*V*_dc_ curves were almost parabolic. In contrast, when a high-frequency AC bias voltage was used, the Δ*f*(*f*_m_)–*V*_dc_ curves were not linear but roughly divided into three regions, and the Δ*f*–*V*_dc_ curves were distorted from a parabolic shape. These differences were due to the dependence on the contribution of the capacitance *C*_it_ caused by the interface states to the electrostatic force. That is, when a low-frequency AC bias voltage is used, the capacitance inside the semiconductor is given by the sum of the capacitance *C*_it_ caused by the interface states and the depletion layer capacitance *C*_D_. In contrast, when a high-frequency AC bias is used, the contribution of the capacitance *C*_it_ due to the interface states is almost negligible, and the depletion layer capacitance *C*_D_ has a large influence on the electrostatic force. In the depletion region, the slope of the Δ*f*(*f*_m_)–*V*_dc_ curve for a low-frequency AC bias was found to be slightly larger than that for a high-frequency AC bias. We demonstrated for the first time that the interface state density *D*_it_ could be estimated from the difference in these slopes.

The experimental result that the Δ*f*–*V*_dc_ curves highly depend on the frequency of the applied AC bias voltage strongly suggests the feasibility of a new spectroscopy method to measure the frequency dependence of carrier transfer in a sample. That is, in this high-low KPFS, only *f*_m_ or 2*f*_0_ + *f*_m_ was used as the frequency of the AC bias voltage, but AC bias voltages with frequencies other than these can be applied to measure Δ*f*–*V*_dc_ curves. Therefore, note that the measurement of the Δ*f*–*V*_dc_ curve can be applied for AC bias voltages with very high frequencies, such as in the microwave region, which are not applicable in the conventional KPFM and KPFS.

In the future, high–low KPFS measurements with 2D scanning of the tip on the sample surface are expected to enable measurement of the local interface state density of the sample surface on the nanoscale. Therefore, the high–low KPFS method proposed in this study is expected to be widely used for sensitive and high-resolution nanoscale measurements of impurity concentration and defect level distributions at the surfaces and interfaces of various semiconductor materials and devices.
